# Offering pre-exposure prophylaxis for HIV prevention to pregnant and postpartum women: a clinical approach

**DOI:** 10.7448/IAS.20.2.21295

**Published:** 2017-03-08

**Authors:** Dominika L Seidman, Shannon Weber, Deborah Cohan

**Affiliations:** ^a^ Obstetrics, Gynecology & Reproductive Sciences, University of California San Francisco, San Francisco, CA, USA; ^b^ Department of Family & Community Medicine, University of California San Francisco, San Francisco, CA, USA

**Keywords:** pre-exposure prophylaxis, pregnancy, lactation, postpartum, HIV prevention, perinatal HIV transmission

## Abstract

**Introduction**: HIV prevention during pregnancy and lactation is critical for both maternal and child health. Pregnancy provides a critical opportunity for clinicians to elicit women’s vulnerabilities to HIV and offer HIV testing, treatment and referral and/or comprehensive HIV prevention options for the current pregnancy, the postpartum period and safer conception options for future pregnancies. In this commentary, we review the safety of oral pre-exposure prophylaxis with tenofovir/emtricitabine in pregnant and lactating women and suggest opportunities to identify pregnant and postpartum women at substantial risk of HIV. We then describe a clinical approach to caring for women who both choose and decline pre-exposure prophylaxis during pregnancy and postpartum, highlighting areas for future research.

**Discussion**: Evidence suggests that pre-exposure prophylaxis with tenofovir/emtricitabine is safe in pregnancy and lactation. Identifying women vulnerable to HIV and eligible for pre-exposure prophylaxis is challenging in light of the myriad of individual, community, and structural forces impacting HIV acquisition. Validated risk calculators exist for specific populations but have not been used to screen and offer HIV prevention methods. Partner testing and engagement of men living with HIV are additional means of reaching at-risk women. However, women’s vulnerabilities to HIV change over time. Combining screening for HIV vulnerability with HIV and/or STI testing at standard intervals during pregnancy is a practical way to prompt providers to incorporate HIV screening and prevention counselling. We suggest using shared decision-making to offer women pre-exposure prophylaxis as one of multiple HIV prevention strategies during pregnancy and postpartum, facilitating open conversations about HIV vulnerabilities, preferences about HIV prevention strategies, and choosing a method that best meets the needs of each woman.

**Conclusion**: Growing evidence suggests that pre-exposure prophylaxis with tenofovir/emtricitabine during pregnancy and lactation is safe and effective. Shared decision-making provides one approach to identify at-risk women and offers pre-exposure prophylaxis but requires implementation research in diverse clinical settings. Including pregnant and breastfeeding women in future HIV prevention research is critical for the creation of evidence-driven public health policies and clinical guidelines.

## Introduction

While a growing body of literature describes safer conception for HIV serodifferent couples [1–3], relatively little focuses on HIV prevention strategies during pregnancy and postpartum. Many women miss opportunities for safer conception: worldwide, 40% of pregnancies are unintended [4], and regions with high rates of unintended pregnancies overlap significantly with high HIV prevalence areas [4]. Furthermore, with persistent stigma around pregnancy and HIV, many couples planning conception do so without consulting healthcare providers [5,6]. Women in serodifferent relationships often initiate antenatal care already pregnant, without the benefit of safer conception counselling [7]. Moreover, for young women, pregnancy may indicate HIV risk for the first time. Therefore, prenatal care provides a critical opportunity to elicit women’s vulnerabilities to HIV. During these visits, healthcare providers can offer HIV testing, treatment and referral and/or comprehensive HIV prevention options for the current pregnancy, postpartum and safer conception options for future pregnancies.

### HIV susceptibility in pregnancy

Limited epidemiologic data suggest that pregnancy is a period of increased HIV susceptibility. Although a meta-analysis of five studies comparing incident HIV in pregnant to non-pregnant women was inconclusive (pooled hazard ratio 1.3, 0.5–2.1), the pooled incidence rate in pregnancy was high (4.7/100 person-years), comparable to other higher risk groups [8]. Biologic data also suggest that pregnancy physiology may increase HIV susceptibility [9–11].

During pregnancy and postpartum, changes in sexual practices including frequency and type of intercourse, condom use and concurrent relationships also affect HIV susceptibility [12–16]. While population-specific data vary, the aggregate demonstrate how sexual practices in pregnancy and postpartum must be regularly assessed to understand women’s current vulnerabilities to HIV.

Violence patterns in pregnancy and postpartum are understudied but may further contribute to HIV vulnerability. Intimate partner violence (IPV) has been associated with HIV acquisition through forced intercourse, inability to negotiate condoms, prolonged stress exposure and increased likelihood of violent partners having multiple HIV risk factors [17]. Although data are conflicting regarding pregnancy’s impact on IPV [18], population-specific IPV prevalence during pregnancy has been reported up to 57% [19,20].

### Incident HIV during pregnancy or lactation

Acute HIV during pregnancy or lactation is associated with increased vertical transmission. In the United States (US), HIV acquisition during pregnancy resulted in 15 times the odds of vertical transmission compared to chronic, treated HIV (aOR 15.2, 95% CI 4.0–56.3) [21] and is responsible for approximately 10% of vertical transmissions [22]. In African cohorts, incident HIV during pregnancy or postpartum was associated with twice the odds of vertical transmission compared with chronic HIV (pooled OR 2.3, 95% CI 1.2–4.4) [8]. Finally, in Zimbabwe, HIV acquired during lactation was associated with a fourfold increase in transmission to breastfed babies compared to breastfed infants of women with chronic, untreated HIV [23].

### Implications for HIV prevention in pregnancy

**Figure 1.**
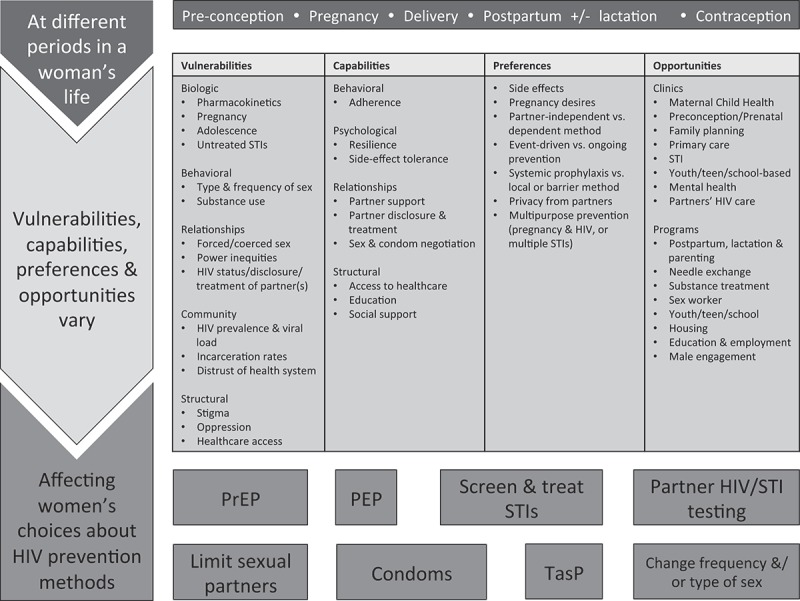
Applying shared decision-making to HIV prevention strategies for reproductive-aged women. A woman’s vulnerabilities to HIV, capabilities, preferences and opportunities vary over time and may change preconception, during pregnancy, and postpartum. Shared decision-making can be used during each of these periods to elicit a woman’s vulnerabilities, capabilities and preferences, facilitating a woman’s choice of HIV prevention method(s) that best meet her current needs. Clinics and programmes provide opportunities to use shared decision-making to offer HIV prevention and support a woman’s chosen method. PEP, post-exposure prophylaxis; PrEP, pre-exposure prophylaxis; STIs, sexually transmitted infections; TasP, treatment as prevention of a partner living with HIV.

Given the importance of HIV prevention during pregnancy and lactation, we first review the safety of oral pre-exposure prophylaxis in pregnant and lactating women. Pre-exposure prophylaxis with tenofovir/emtricitabine is a highly effective HIV prevention tool that does not rely on partner participation and facilitates protection before exposure, providing a critical new option for women [24]. We then discuss opportunities to identify pregnant and postpartum women at substantial risk of HIV. Finally, we suggest clinical care recommendations including shared decision-making to offer women pre-exposure prophylaxis as one of multiple HIV prevention strategies (Figure 1). Due to limited data on pre-exposure prophylaxis in pregnancy and postpartum, we review evidence from diverse settings with variable resources, acknowledging that implementation will require testing in local settings.

## Discussion

### Efficacy and safety of oral pre-exposure prophylaxis with tenofovir/emtricitabine in pregnancy and lactation

Pre-exposure prophylaxis is highly effective at preventing HIV in non-pregnant women. A meta-analysis of 18 studies reported a relative risk for HIV acquisition comparing pre-exposure prophylaxis with placebo of 0.54 (95% CI: 0.32–0.90) for penile/vaginal exposures and 0.34 (95% CI: 0.15–0.80) for rectal exposures, without differences by sex [25]. The Partners Demonstration Project preliminarily described 96% effectiveness of pre-exposure prophylaxis when used as a bridge to treatment as prevention in over 1000 serodifferent Kenyan and Ugandan couples [26]. Vaginal protection requires high adherence [27]; how protective levels vary during pregnancy and lactation is unknown.

Several pre-exposure prophylaxis studies included pregnant women, although the majority required stopping therapy at pregnancy diagnosis [1]. A Partners PrEP sub-study described 431 women who incidentally became pregnant while taking pre-exposure prophylaxis; the latest exposure was at 7 weeks gestation. No differences in pregnancy loss, preterm birth, congenital anomalies and one year of postnatal growth were detected [28]. A retrospective review of 27 women in the US who were offered pre-exposure prophylaxis preconception, during pregnancy and postpartum, found no adverse pregnancy outcomes in women who used tenofovir/emtricitabine [29].

There are robust safety data on tenofovir for pregnant women living with HIV or hepatitis B. The Antiretroviral Pregnancy Registry suggests that tenofovir/emtricitabine is safe in pregnancy, reporting enough data to detect a 1.5-fold increase in anomalies with first trimester exposures [30]. The World Health Organization (WHO), the US Department of Health and Human Services and the American Association for the Study of Liver Diseases recommend tenofovir-based therapy for pregnant women living with HIV or hepatitis B [31–34]. A systematic review of tenofovir use in pregnancy (26 studies of women with HIV and 5 of women with hepatitis B) found no differences in pregnancy loss, preterm birth (<37 weeks), low birth weight infants (<2500 g and <1500 g), small for gestational age infants, birth defects and infant or maternal mortality [35].

While safety data on tenofovir exposure and most birth outcomes are robust, unanswered questions remain about infant bone mineral content and postnatal growth. One study reported a clinically insignificant decrease (<0.5 cm) in infant height and head circumference at one year among in-utero, tenofovir-exposed infants born to women living with HIV [36], but this result has not been replicated [37–39]. Furthermore, while one study found a 12% decrease in bone mineral content among 74 in-utero HIV and tenofovir-exposed infants compared to 69 infants exposed to other antiretrovirals [40], a trial of 425 infants suggested an association between decreased bone mineral content and *any* triple therapy, not specifically tenofovir [41].

Pharmacokinetic studies suggest that infant tenofovir/emtricitabine exposure is lower through breast milk than in utero [42,43]. A study of 50 HIV-negative breastfeeding women receiving tenofovir/emtricitabine found that tenofovir was not detected in 94% of infant serum samples. Emtricitabine was detectable in 96% of samples, but the estimated equivalent dose an infant would ingest daily from breastfeeding was 0.5% of the infant HIV treatment dose [44].

Highly relevant for postpartum women, the Partners PrEP Study suggested no interactions between hormonal contraceptives and pre-exposure prophylaxis, and no change in the effectiveness of HIV or pregnancy prevention [45,46].

Based on these data, the WHO and US Centers for Disease Control and Prevention suggest offering pre-exposure prophylaxis during pregnancy and lactation, discussing risks and benefits with women [47,48]. The American College of Obstetricians and Gynecologists acknowledges tenofovir/emtricitabine’s ‘reassuring’ safety profile in pregnancy, and states that clinicians should be ‘vigilant’ for HIV seroconversion during lactation [49]. Finally, a decision analysis of pre-exposure prophylaxis use in pregnant and lactating African women found that even when accounting for possible increases in preterm births, pre-exposure prophylaxis is likely cost-effective [50].

### Identifying women vulnerable to HIV in pregnancy and lactation

The WHO recommends offering pre-exposure prophylaxis to individuals at substantial risk of HIV as part of combination HIV prevention services. Data on how to operationalize these recommendations are growing but are particularly nascent for pregnant and postpartum women.

Validated risk scores exist for African women who know and do not know their male partner(s)’ HIV status. These calculators include women’s demographics, sexual practices, sexually transmitted infections (STIs – if available) and partner factors (viral load, sexual practices and whether he provides financial/material support) [51,52]. Notably, one score requires awareness of partner status; one score was developed in exclusively young, at-risk women; and the majority of women in both studies were required to use contraception. Whether these calculators are applicable to pregnant and lactating women, as well as women with other demographics, is unknown.

One study in Kenya reports predictors of HIV seroconversion during pregnancy including current syphilis (HR 9.18, 95% CI 2.15–39.3), chlamydia (HR 4.49, 95% CI 1.34–15.0), yeast vaginitis (HR 3.46, 95% CI 1.46–8.19), bacterial vaginosis (HR 2.91, 95% CI 1.25–6.76) and prior STIs (HR 3.48, 95% CI 1.31–9.27). Strikingly, none of the women who acquired HIV reported having a partner living with HIV, while no women who disclosed having a partner living with HIV seroconverted [53]. While many regions rely on syndromic STI management, this study highlights the utility of integrating STI testing into antenatal and postpartum care to identify women vulnerable to HIV.

Risk calculators provide an appealing approach to efficiently identify women vulnerable to HIV but have not been used to screen and offer women pre-exposure prophylaxis. In lower HIV prevalence regions where risk calculators are not available, clinicians often piece together epidemiologic data to assess risk. For example, in the US, while African-Americans comprise 16% of the population, they account for 64% of new HIV diagnoses in women. Even with the same number of sexual partners and encounters, African-American women are at higher risk of HIV acquisition [54,55]. Additional factors associated with HIV diagnoses in US women include recent gonorrhoea or syphilis [56], IPV [57], exchange sex and drug use [58], among others. However, many US women diagnosed with HIV had no identifiable risk factor other than heterosexual sex [56].

Studies from higher and lower prevalence settings highlight the myriad of forces impacting women’s vulnerabilities to HIV and demonstrate how identifying at-risk women requires multifaceted assessments of individual, community and structural determinants [24]. In addition, factors affecting HIV susceptibility change over time. The optimal time(s) to assess HIV vulnerability in pregnancy is not known, but a logical approach is to link assessment with HIV and STI testing [29]. Since universal prenatal HIV and syphilis testing is recommended at least once, performing testing and vulnerability assessments together may increase clinicians’ screening practices. Maintaining heightened vigilance postpartum is particularly challenging given a lack of standardized HIV testing recommendations, coupled with high postpartum loss-to-follow-up rates. Incorporating HIV vulnerability assessments into postpartum, paediatric and family planning visits may identify women who present for only one type of care.

Partner testing and engagement remain cornerstones of identifying women vulnerable to HIV. Several studies describe partner HIV testing of HIV-negative pregnant women in antenatal clinics and off site [59–65]. The HOPE trial in Kenya found that home-based testing for pregnant women and their partners was cost-effective [66]. Furthermore, qualitative research from Partners PrEP suggests that supportive partners promote, while relationship discord impedes, pre-exposure prophylaxis adherence [67]. Male partner involvement has been associated with prevention of vertical transmission in women living with HIV [68]; whether male involvement enhances pre-exposure prophylaxis adherence during pregnancy and lactation is unknown, but plausible.

Reaching pregnant and lactating women through male partners living with HIV may be an additional means of identifying women. However, limited data suggest that this approach is underutilized: in a survey of providers of men living with HIV in San Francisco, 25% never asked male patients about fertility desires, and half had ever seen a couple together [69]. HIV care providers’ offering prevention methods to seronegative partners and couple-based models of care may be efficient but require provider training. Moreover, this approach is limited to identifying women whose partners have undergone testing, engaged in care and disclosed their HIV status.

### A clinical approach to offering HIV prevention options to pregnant and postpartum women

The challenges of identifying women at substantial risk of HIV highlight the importance of educating *all* women about available HIV prevention methods. Periods of vulnerability shift [29]; many women engage in healthcare exclusively during pregnancy, allowing opportunities to expand community knowledge of HIV prevention including pre-exposure prophylaxis. Shared decision-making provides a framework for identifying vulnerable women and offering prevention strategies that best meet women’s needs (Figure 1) [24].

In a clinical encounter structured by shared decision-making, the patient reviews her vulnerabilities to HIV, while the clinician provides evidence-based information; elicits patient experiences, values and preferences and helps the patient weigh competing priorities. Together, patient and provider arrive at a preferred choice [70]. Shared decision-making is best applied to clinical scenarios where there are multiple options and no clear recommendation. For HIV prevention, options include pre-exposure prophylaxis, post-exposure prophylaxis, partner(s’) testing, treatment of partner(s) living with HIV as prevention, condom use, altering sexual practices and STI testing and treatment.

Although shared decision-making may seem implausible in low-resource settings, experience from resource-rich settings suggests opportunities to increase efficiency and quality of counselling, particularly through use of decision-support tools and task sharing [71]. Equally importantly, data on African women’s preferences for HIV prevention counselling support the use of shared decision-making [72].

While not all providers are comfortable counselling about nuances of each HIV prevention method in pregnancy, all providers in contact with pregnant and breastfeeding women should be familiar with post-exposure prophylaxis due to time-sensitive eligibility (within 72 h of exposure). Post-exposure prophylaxis is safe in pregnancy and lactation [73] and may provide a useful bridge while women await referrals to providers who offer comprehensive care.

Caring for pregnant and postpartum women vulnerable to HIV necessitates regular assessment of changing HIV vulnerabilities, satisfaction with chosen prevention methods and adherence. While there are no studies on adherence support for pre-exposure prophylaxis in pregnancy and postpartum, data may be extrapolated from outside of pregnancy and HIV treatment programmes [74,75]. Tailoring support to particularly vulnerable groups – adolescents, women affected by violence, women who inject drugs and postpartum women – is a critical area for future research.

There are no published protocols for laboratory monitoring of pregnant or breastfeeding women vulnerable to HIV, whether or not they are using pre-exposure prophylaxis. Furthermore, there is no guidance on monitoring male partners living with HIV during HIV-negative women’s pregnancies and lactation. Additional testing of women most vulnerable to HIV is prudent in pregnancy and lactation to detect acute seroconversion. However, the frequency and type of testing depend on prevention method(s) used, frequency of HIV exposure and local resources. Ideally, a man living with HIV with an HIV-negative pregnant or breastfeeding partner would have frequent viral load and STI monitoring, with results shared with the woman’s provider. Practices will necessarily vary due to resource constraints and absence of guidelines.

Even fewer data are available to guide postpartum care. A woman’s capabilities, values and preferences may change postpartum, necessitating reassessment of vulnerabilities to HIV and prevention choices (Figure 1). Data from women living with HIV indicate that antiretroviral adherence frequently decreases after delivery, and women benefit from additional support [76–78]. Consequently, postpartum-specific research is needed to ensure women remain HIV free for their long-term health, prevention of lactational transmission and potential future pregnancies. Integrating postpartum, family planning, women’s health and paediatric care may facilitate reaching women who are lost to follow-up after birth.

## Conclusions

HIV prevention strategies during pregnancy and postpartum are paramount to maintaining maternal health, eliminating vertical transmissions and facilitating safer conception for future pregnancies. Growing evidence suggests that pre-exposure prophylaxis during pregnancy and lactation is a safe and effective HIV prevention option. Shared decision-making is one approach to identifying at-risk women and offering pre-exposure prophylaxis but requires implementation research in diverse clinical settings. Including pregnant and breastfeeding women in HIV prevention studies is critical for development of evidence-driven public health policy and clinical guidelines.
